# Pros and Cons of the Tuberculosis Drugome Approach – An Empirical Analysis

**DOI:** 10.1371/journal.pone.0100829

**Published:** 2014-06-27

**Authors:** Feng-Chi Chen, Yu-Chieh Liao, Jie-Mao Huang, Chieh-Hua Lin, Yih-Yuan Chen, Horng-Yunn Dou, Chao Agnes Hsiung

**Affiliations:** 1 Division of Biostatistics and Bioinformatics, Institute of Population Health Sciences, National Health Research Institutes, Zhunan, Miaoli County, Taiwan; 2 Department of Life Sciences, National Chiao-Tung University, Hsinchu, Taiwan; 3 Department of Dentistry, China Medical University, Taichung, Taiwan; 4 Institute of Bioinformatics and Structural Biology, National Tsing Hua University, Hsinchu, Taiwan; 5 National Institute of Infectious Diseases and Vaccinology, National Health Research Institutes, Zhunan, Miaoli County, Taiwan; Johns Hopkins University School of Medicine, United States of America

## Abstract

Drug-resistant *Mycobacterium tuberculosis* (MTB), the causative pathogen of tuberculosis (TB), has become a serious threat to global public health. Yet the development of novel drugs against MTB has been lagging. One potentially powerful approach to drug development is computation-aided repositioning of current drugs. However, the effectiveness of this approach has rarely been examined. Here we select the “TB drugome” approach – a protein structure-based method for drug repositioning for tuberculosis treatment – to (1) experimentally validate the efficacy of the identified drug candidates for inhibiting MTB growth, and (2) computationally examine how consistently drug candidates are prioritized, considering changes in input data. Twenty three drugs in the TB drugome were tested. Of them, only two drugs (tamoxifen and 4-hydroxytamoxifen) effectively suppressed MTB growth at relatively high concentrations. Both drugs significantly enhanced the inhibitory effects of three first-line anti-TB drugs (rifampin, isoniazid, and ethambutol). However, tamoxifen is not a top-listed drug in the TB drugome, and 4-hydroxytamoxifen is not approved for use in humans. Computational re-examination of the TB drugome indicated that the rankings were subject to technical and data-related biases. Thus, although our results support the effectiveness of the TB drugome approach for identifying drugs that can potentially be repositioned for stand-alone applications or for combination treatments for TB, the approach requires further refinements via incorporation of additional biological information. Our findings can also be extended to other structure-based drug repositioning methods.

## Introduction

Tuberculosis (TB) is one of the most serious threats to global public health. In 2011 alone, there were 8.7 million new cases of TB infection and 1.4 million TB-related deaths, according to the 2012 World Health Organization (WHO) Global Tuberculosis Report. Difficulties in treating TB lie partly in the emergence of drug-resistant strains of *Mycobacterium tuberculosis* (MTB), the major causative pathogen of TB. Particularly, multidrug-resistant MTB strains, those that are resistant to the first-line drugs rifampin (RIF) and isoniazid (INH), have been circulating for years [1]. Recently, extensively drug-resistant MTB strains (those that are resistant to INH and RIF, plus any fluoroquinolone and at least one of three injectable second-line drugs) have been identified in many countries [2], further escalating the challenges of controlling TB [3].

The development of novel TB treatments has been slow, despite the severity of the disease in global health. Given the high cost of developing new drugs, researchers have been trying to reposition existing drugs to treat TB [4]. An innovative computational approach was recently proposed to reposition currently approved drugs to treat TB [5], [6]. This “TB drugome approach,” if proven feasible, will markedly accelerate the process of MTB drug development. The TB drugome approach incorporates structural bioinformatics, molecular modeling, and protein-drug interaction network analyses to predict potential MTB drugs, on the basis of the known protein targets of approved human drugs and the similarities between the three-dimensional structures of MTB proteins and human proteins. Drugs identified with this method are collectively termed the “TB drugome” [5]. Although the prediction results appear to be promising, the efficacy of the set of predicted drugs has yet to be experimentally validated.

In addition to predicting stand-alone drugs for TB treatment, the TB drugome approach can potentially be used to identify drugs for combination treatments, a proven strategy to tackle drug resistance [7]. The rationale behind this strategy is that different drugs attack different MTB targets, which are unlikely to mutate and develop drug resistance simultaneously. Combining two or more drugs to treat TB might not only decrease the probability of drug resistance, but also increase the effectiveness and shorten the duration of treatment regimens [7]. These advantages are particularly important in light of the long treatment regimens and low patient compliance of traditional TB treatments [8], [9].

In this study, we conducted an updated TB drugome analysis, including protein structural information from the RCSB Protein Data Bank (PDB) as of January 2013 following the procedure described by Kinnings *et al.* [5]. We compared the updated TB drugome with the original one, and experimentally examined the effects of the top candidates in the TB drugome on the growth of MTB strains H37Rv and H37Ra. Despite inconsistencies between the original and updated drugomes, two of the tested drugs, alone or in combination with first-line anti-TB drugs, effectively suppressed the growth of both MTB strains. Therefore, we provide experimental evidence that a computation-based rational approach to designing novel TB treatment is feasible. We also discuss the limitations of and potential improvements to the computational approach.

## Results

### Re-examination of the TB drugome

Following the methods published by Kinnings *et al*. [5], we obtained a new TB drugome using updated structural information for drug targets and MTB proteins. Of the 311 included human drugs, 217 had at least one potential MTB protein target. On average, each drug had ∼6.6 (2053/311) potential MTB target proteins. However, the number of potential target proteins was unevenly distributed across the 217 drugs, with 75 drugs having only one and 62 drugs having more than 10 potential MTB targets (Figure S1). Most of the drugs that Kinnings *et al.* included in their list of top-15 hits also appeared in our top list, although some of them had different rankings (e.g., RIF, amantadine, propofol, ritonavir, lopinavir, penicillamine, and nelfinavir; Table 1). This observation suggests that the drugs in the “top list” vary based on the availability of protein structural information and may be somewhat biased.

**Table 1:** List of the drugs tested in this study.

Drug	Chemical component identifier	Rank		No. of potential proteins			CAS^c^ number	Plasma concentration^e^
		Kinnings *et al.*	This study	Kinnings *et al.*		This study		(mg/L)
				H+S^a^	S only^b^			
Isotretinoin	REA	1	1	98	14	81	4759-48-2	0.3–0.5 [29]
Levothyroxine	T44	2	2	63	14	73	25416-65-3	4.4–6.4 [30]
Methotrexate	MT1+MTX	3	2	48	10	73	133073-73-1	0.3–6.4 [31]
Estradiol	EST	4	5	38	10	63	50-28-2	3.5–10 E-5 [32]
Rifampin	RFP	5	24	34	6	24	13292-46-1	8.0–11.1 [33]
4-hydroxytamoxifen^d^	OHT	6	4	33	10	66	68392-35-8	4.0–73 E-4 [14]
Amantadine	308	7	57	32	0	12	665-66-7	0–1.65 [34]
Raloxifene	RAL	8	7	28	10	53	82640-04-8	3.0–11 E-4 [35]
Propofol	PFL	9	48	24	3	15	2078-54-8	1.9 [36]
Indinavir	MK1	10	16	23	2	31	150378-17-9	0.02–7.1 [37]
Ritonavir	RIT	11	22	22	7	25	155213-67-5	13.4–33.3 [38]
Darunavir	017	11	6	22	5	54	206361-99-1	1.8–12.9 [39]
Lopinavir	AB1	11	39	22	4	18	192725-17-0	3.6–8.9 [40]
Penicillamine	LEI	14	26	20	5	23	52-67-5	0.6–1.0 [41]
Nelfinavir	1UN	14	45	20	3	16	159989-65-8	0.1–11.7 [42]
Dexamethasone	DEX		15	10	2	32	50-02-2	1.2–8.4 E-3 [43]
Fluconazole	TPF		>100	NA	NA	3	86386-73-4	1.9–6.7 E-3 [44]
Trimethoprim	TOP+TRR		30	NA	NA	21	738-70-5	4.0–10.5 E-3 [45]
Cytarabine	AR3+CTN		75	NA	NA	6	147-94-4	0.9–5.5 [46]
Spironolactone	SNL		26	3	2	23	52-01-7	0.1–0.6 [47]
Indomethacin	IMN		13	10	3	33	53-86-1	2.6–3.1 [48]
Liothyronine	T3		33	3	1	20	6893-02-3	3.0–7.0 E-6 [49]
Progesterone	STR		24	3	2	24	57-83-0	0.2–11.4 [50]
Tamoxifen	CTX		>100	NA	NA	1	10540-29-1	0.16–0.49 [26]

a Ranking was based on homology-based structural predictions and experimentally determined structures of MTB proteins.

b Ranking was based solely on experimentally determined structures of MTB proteins.

c Chemical Abstracts Service.

d 4-hydroxytamoxifen is not an FDA-approved human drug.

e Plasma concentrations of a drug may vary significantly between experiments because of biological variations and differences in dosing scheme, experimental design, and drug-detection technology. For simplicity, here we selected only one reference for each drug. For hormone replacement therapies (e.g., estradiol, levothyroxine, liothyronine, and progesterone), the plasma concentration may indicate the concentration of the hormone of interest with or without administration of the therapy. Also, concentrations in the reference studies may have been reported in units other than mg/L (e.g., nmol/L). In such cases, the units were converted to mg/L.

### Experimental validation of the efficacy of the TB drugome

We experimentally validated whether the drugs identified in the top list by the TB drugome inhibited the growth of MTB. We selected 23 drugs (Table 1) and tested them against the H37Ra MTB strain. Most of these drugs were top candidates in the original TB drugome. The original and the updated TB drugomes both identified 4-hydroxytamoxifen (4-OHT), which is not an FDA-approved drug but rather a derivative of another approved drug, tamoxifen. Hence, we also included tamoxifen in this experiment.

**Figure 1:**
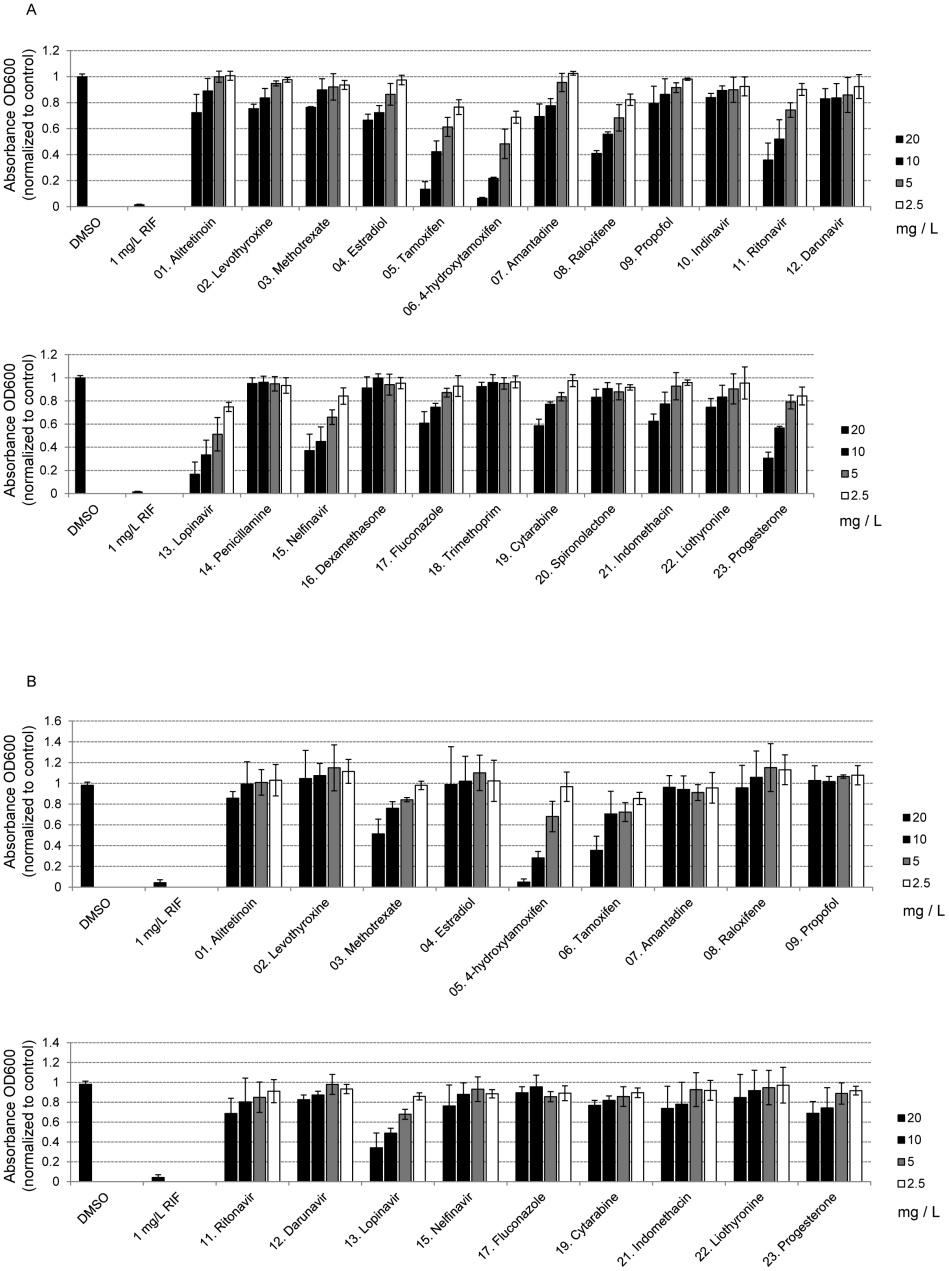
Inhibitory effects of the 23 tested chemicals on (A) *M. tuberculosis* H37Ra and (B) and *M. tuberculosis* H37Rv.

As shown in Figure 1A, although the candidate drugs were less effective than the first-line drug RIF, some of them were able to inhibit the growth of *M. tuberculosis* H37Ra at the tested concentrations. Drugs that showed a concentration-dependent inhibitory effect included alitretinoin (#01), levothyroxin (#02), methotrexate (#03), estradiol (#04), tamoxifen (#05), 4-OHT (#06), amantadine (#07), raloxifene (#08), ritonavir (#11), lopinavir (#13), nelfinavir (#15), fluconazole (#17), cytarabine (#19), indomethacin (#21), and progesterone (#23). Interestingly, many of these drugs are either antiviral drugs (ritonavir and nelfinavir) or sex hormone-related drugs (estradiol, tamoxifen, 4-OHT, raloxifene, and progesterone).

At a concentration of 20 mg/L, 17 of the 23 tested drugs significantly inhibited the growth of *M. tuberculosis* H37Ra. Exceptions included alitretinoin (#01), propofol (#09), penicillamine (#14), dexamethasone (#16), trimethoprim (#18), and spironolactone (#20). However, 20 mg/L is much higher than the clinically relevant concentration for most of these drugs (Table 1). Therefore, even though these drugs repressed MTB growth, at such a high concentration, they are likely intolerable to the human body.

In comparison, at the lowest concentration tested (2.5 mg/L), only four of the drugs significantly repressed the growth of *M. tuberculosis* H37Ra, including tamoxifen (#05), 4-OHT (#06), raloxifene (#08), and lopinavir (#13). The minimal inhibitory concentration values (MIC_50_ and MIC_90_ values) of the tested chemicals were inferred with reference to the growth inhibition experiments. The MIC_50_ and MIC_90_ values of most of the tested chemicals could not be determined (designated as “ND”) because they were beyond the highest tested concentration (20 mg/L; Table S1). Nevertheless, the approximate MIC_50_ values could be determined for tamoxifen (5–10 mg/L), 4-OHT (2.5–5 mg/L), raloxifene (10–20 mg/L), ritonavir (10–20 mg/L), lopinavir (5–10 mg/L), nelfinavir (5–10 mg/L), and progesterone (10–20 mg/L). The approximate MIC_90_ could only be determined for 4-OHT (10–20 mg/L).

*M. tuberculosis* H37Rv is slightly different genetically from the avirulent strain H37Ra. Therefore, we examined whether the same drugs could effectively inhibit the growth of H37Rv. We excluded five drugs that had little or no inhibitory effect on H37Ra (indinavir, penicillamine, dexamethasone, trimethoprim, and spironolactone) and tested the remaining 18 drugs against H37Rv. As shown in Figure 1B, some of the drugs that were effective against H37Ra were ineffective against H37Rv, even at the highest tested concentration (20 mg/L). These drugs included levothyroxin (#02), estradiol (#04), amantadine (#07), raloxifene (#08), ritonavir (#11), nelfinavir (#15), fluconazole (#17), indomethacin (#21), and liothyronine (#22).

Only four of the remaining drugs demonstrated a clear concentration-dependent inhibitory effect: methotrexate (#03), tamoxifen (#05), 4-OHT (#06), and lopinavir (#13). However, the lowest concentration at which these drugs had a significant inhibitory effect on H37Rv was 5 mg/L, which was higher than what was observed for H37Ra. The approximate MIC values were as follows: tamoxifen, MIC_50_ 10­–20 mg/L; 4-OHT, MIC_50_ 5–10 mg/L, MIC_90_ 10–20 mg/L; and lopinavir, MIC_50_ 5–10 mg/L. For all other drugs, the MIC_50_ and MIC_90_ values could not be determined (Table S2). These observations suggest that the virulent strain H37Rv was able to tolerate a higher drug concentration than the avirulent strain H37Ra, despite the similarities in their genetic backgrounds.

To examine whether 4-OHT was able to inhibit clinical isolates of MTB, we had 4-OHT tested against two isolates (0107-37 and 0104-12) alongside H37Ra and H37Rv, using the MGIT (Mycobacteria Growth Indicator Tube) broth dilution method at the Tri-Service General Hospital of Taiwan. The concentrations to completely inhibit the growth of H37Ra, H37Rv, 0107-37, and 0104-12, were 20, 20, 40, and 20 mg/L, respectively. These concentrations were similar to what we had previously observed (Tables S1 and S2). Despite this difference, two independent laboratories demonstrated an *in vitro* inhibitory effect of 4-OHT on MTB growth.

Tamoxifen and 4-OHT were particularly potent inhibitors of both MTB strains (H37Ra and H37Rv). This result was somewhat surprising because tamoxifen, with only one predicted MTB target, was not in the top-100 candidates. This observation suggests that the ranking system of the TB drugome approach can be further refined.

### Effect of drug combinations on MTB growth

We examined the effects of combination treatment using 4-OHT and one of the three first-line TB drugs (RIF, INH, and ethambutol [EMB]) against the H37Ra strain. Three to five different concentrations of the first-line drugs and three concentrations of 4-OHT were tested. Concentrations selected for INH and EMB were serial dilutions starting from the MIC_50_ (0.05 mg/L for INH and 1.6 mg/L for EMB), as previously reported [10]. For RIF, the selected concentrations represented the concentration that completely inhibited MTB growth (MaxC: 1.56 E-2 mg/L), 1/4 MaxC (3.90 E-3 mg/L), 1/16 MaxC (9.70 E-4 mg/L), and a lower concentration that had only marginal inhibitory effect (1.2 E-4 mg/L), based on the growth curve of RIF-treated H37Ra generated in our laboratory (Figure S2). Concentrations of 4-OHT were selected on the basis of the MGIT test results from the Tri-Service General Hospital: the MIC_50_ (20 mg/L), 1/2 MIC_50_ (10 mg/L), and 1/4 MIC_50_ (5 mg/L) effective against H37Ra and H37Rv.

**Figure 2:**
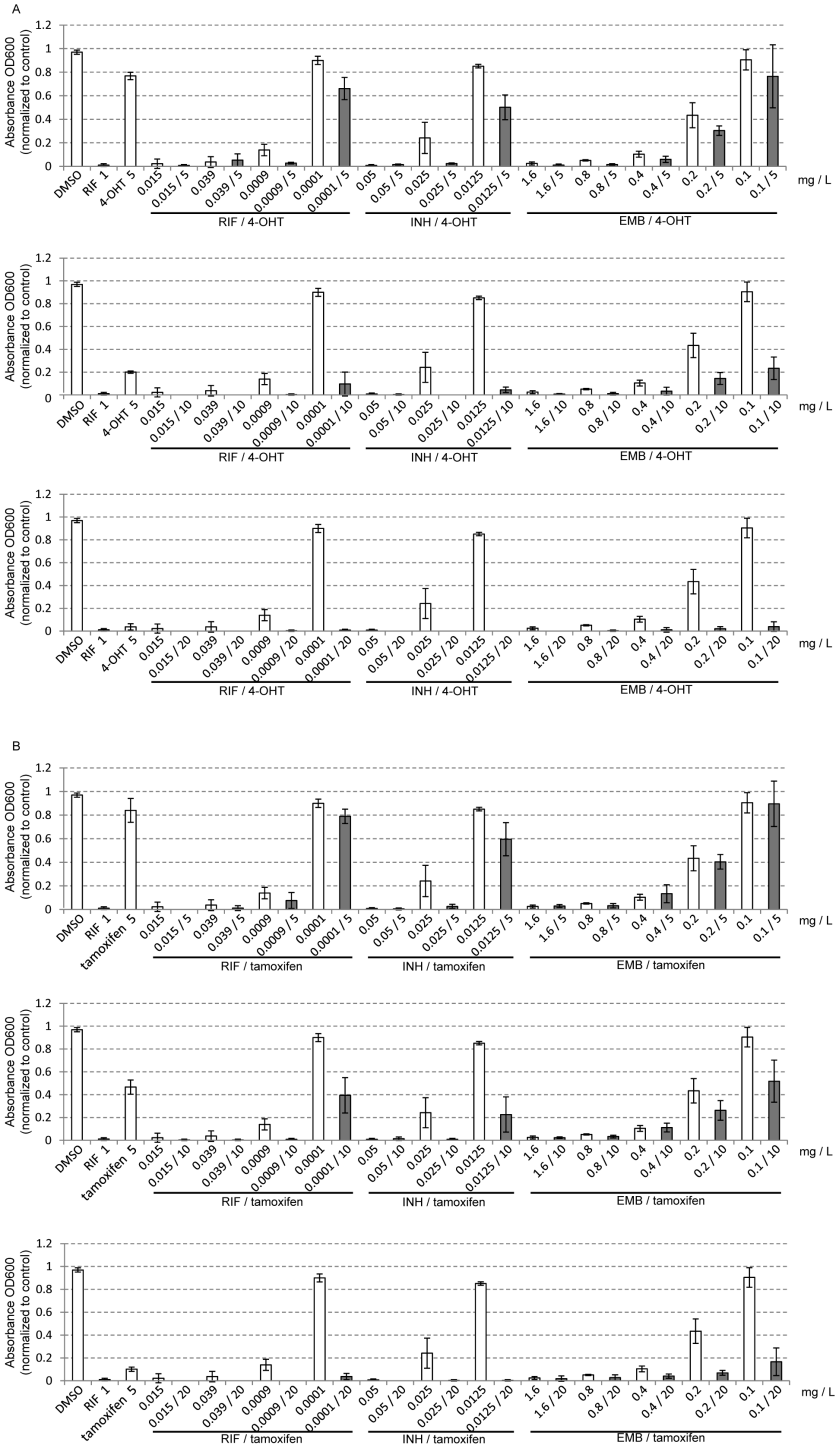
Inhibitory effects of drug combinations on *M. tuberculosis* H37Ra. (A) RIF/INH/EMB plus 4-OHT; (B) RIF/INH/EMB plus tamoxifen. RIF: rifampin; INH: isoniazid; EMB: ethambutol; 4-OHT: 4-hydroxytamoxifen.

Adding the lowest concentration of 4-OHT (5 mg/L) had no significant influence on the inhibitory effects of the three first-line drugs (Figure 2A and Table S3). However, medium (10 mg/L) and high concentrations (20 mg/L) of 4-OHT significantly enhanced the inhibitory effects of all three drugs. In some cases, there were no detectable bacteria in the medium. A similar experiment conducted with tamoxifen produced similar results (Figure 2B and Table S4). Overall, our results suggest that 4-OHT and tamoxifen could potentially be used in combination treatments for TB, and that the TB drugome approach could be applied to identify other drugs for cocktail treatments.

## Discussion

In this study, we experimentally validated part of the computationally predicted TB drugome. We identified two drugs that were effective in inhibiting the growth of MTB strains. Although our results support applying the TB drugome approach to identify potential TB drugs, they also suggest that this approach requires further improvements.

First, only two of the top-15 drugs proved to be effective: 4-OHT and the known first-line drug, RIF. The rate of true-positive prediction was ∼13% (2/15). However, given that the purpose was to identify repositioned drugs, RIF should be excluded (because it is a known anti-TB drug), which decreases the true-positive prediction rate to ∼7% (1/14). Although low-ranked among the included drugs, tamoxifen had inhibitory effects on MTB growth. Thus, the ranking system of the TB drugome appears to be inappropriate for prioritizing drug candidates. Moreover, the 7% hit rate for the TB drugome approach is not very impressive when compared with other approaches. For example, kinase inhibitor library screening studies have yielded hit rates ranging between 0.14% [11] and 5.2% [12]. Bayesian machine learning models can significantly increase the hit rate of high-throughput screening (HTS) for anti-TB drugs to approximately 15–28% [13]. The Bayesian approach was built based on a large quantity of experimental data (HTS results, growth inhibitory concentrations, and cytotoxicity data). Indeed, the accuracy of computational predictions can generally be improved, given sufficient experimental data for model training and validation. The TB drugome approach would likely also be improved if more experimental information were to be incorporated, as discussed below.

Second, the rankings provided by the TB drugome approach varied with the available protein structural data (Table 1). We utilized 1,729 drug-target entries and 1,215 MTB protein entries from the RCSB PDB, compared with 962 and 749 entries respectively, in the original drugome study. The original study used a homology-based approach to predict the structures of MTB proteins, which was not used in this study. Thus, it is not surprising that the rankings generated by these two studies differed significantly from each other (Table 1). In other words, the ranking system is subject to data bias.

Third, for the drugs that demonstrated inhibitory effects, the required concentration was too high for clinical administration (Figures 1 and 2; Table 1). For example, breast cancer patients receiving 20 mg/day of tamoxifen have plasma tamoxifen and 4-OHT concentrations of approximately 0.009–0.134 mg/L and 0–0.007 mg/L, respectively [14]. In this study, the MIC_50_ values of tamoxifen and 4-OHT against MTB H37Rv were 10–20 mg/L and 5–10 mg/L, respectively, which are much higher than can be obtained with current clinically relevant dosing. Thus, this treatment will only be clinically viable after the tamoxifen toxicity has been reduced through chemical modifications.

Animal models have shown that the effectiveness of a drug in an animal is influenced by several factors, such as the dosing scheme, bioavailability, and biological variations (e.g., genetic backgrounds) between individuals. Furthermore, the plasma concentration is not equal to the concentration in the target tissue. Tamoxifen tends to concentrate in tissues. For instance, the hepatic and muscle concentrations of tamoxifen in mice are 160 and 8 times the plasma concentration, respectively [15]. In light of the high tamoxifen/4-OHT concentrations required to inhibit MTB growth, hepatotoxicity is possible if these drugs were to reach MTB-inhibitory concentrations in a mouse.

The low predictive accuracy of the TB drugome approach may be owing to the omission of pharmacokinetics from the method. The model does not consider whether MTB is permeable to the candidate drugs, nor does it consider the rate of uptake for each drug by MTB. The inhibitory effects of candidate drugs would be significantly reduced if they could not penetrate the MTB cells. Binding affinities between candidate drugs and the predicted target proteins were also not evaluated. Furthermore, the biological importance of the target proteins should be considered. For instance, a drug that can bind multiple MTB proteins with low affinities would have only minimal inhibitory effects, even though it is ranked among the top candidates on the TB drugome list. In contrast, a drug that binds and blocks an essential MTB protein is expected to be more effective than one that blocks multiple unimportant proteins. These are some examples reflecting how the TB drugome ranking system does not work well. Another possible area of improvement for this approach is to incorporate pathway information. A drug, or combinations of drugs, that can simultaneously block multiple pathways essential for MTB growth/survival would probably be more effective than a drug that cannot block such pathways.

Finally, the mechanism underlying the inhibitory effects of the candidate drugs remains elusive. Although the TB drugome approach predicts that the candidate drugs can bind and block the target MTB proteins, whether this prediction is biologically true remains unknown. This issue is further complicated by the fundamental differences between human and bacterial proteins. Human proteins usually are post-translationally modified, have multiple isoforms, and form protein families. These features are generally inapplicable to bacteria. How these differences affect drug-protein interactions needs to be clarified to increase the accuracy of the drugome approach.

The combined use of 4-OHT with INH or EMB effectively suppressed MTB growth. One important issue to consider for these combinations is potential drug-drug interactions. 4-OHT was previously found to inhibit the activity of CYP3A (cytochrome P450 family 3, subfamily A) [16], an important drug-metabolizing enzyme, suggesting that 4-OHT could affect the metabolism and activities of other anti-TB drugs. However, experiments in mice showed that CYP3A has no effect on the systemic pharmacokinetics of INH [17]. The observation that both tamoxifen and EMB may cause retinopathy suggests that there could be a possible link between 4-OHT and EMB [18]. However, evidence for direct interactions between the two drugs has not been reported.

Although we found no evidence suggesting interactions between 4-OHT and INH or EMB, other drug interaction mechanisms may exist. Tamoxifen and 4-OHT can activate the pregnane X receptor and indirectly affect its downstream enzyme, CYP3A [19], which is also induced by RIF. This finding appears to suggest that tamoxifen/4-OHT and RIF share certain molecular targets or target structures. The TB drugome approach predicted that tamoxifen could target thymidylate kinase (Rv3247c), which is included in the list of 66 proteins targeted by 4-OHT (Table S5; note that some targets are redundant). Interestingly, the 24 predicted targets of RIF also included thymidylate kinase (Table S6). This observation implies that tamoxifen/4-OHT and RIF may share a similar molecular mechanism for inhibiting MTB growth. This hypothesis and its implications for drug-drug interactions remain to be experimentally validated.

One potential limitation of this study is that we evaluated MTB growth based on the OD_600_ method that, although commonly used in bacteria-related studies [20], [21], may vary with biological conditions [22] (e.g., alterations in the cell wall). To validate our OD_600_ measurements, we compared MTB growth inhibition measured by the OD_600_ method and a fluorescence-based ELISA (enzyme-linked immunosorbent assay) in the presence of different concentrations of INH and EMB. In the ELISA approach, the *zsgreen* green fluorescence protein (GFP) reporter gene was transformed into *M. tuberculosis* H37Ra via the pMV261 vector. An excitation wavelength of 495 nm was applied for ELISA detection of the 525 nm green fluorescence from living TB cells. The two measurements were generally consistent with each other (R^2^ = 0.87 for both INH and EMB; Figure S3). Thus, the level of accuracy of the OD_600_ measurements used in this study seems to be acceptable.

Despite these caveats, the TB drugome approach appears to be a potentially useful method for the identification of novel drugs. It can significantly reduce the searching space for drugs for further experimental validation. In the current drugome approach, only FDA-approved drugs were analyzed (except for 4-OHT). The analysis can easily be extended to include chemicals similar to approved drugs and even to novel compounds. Including chemoinformatics [23], pharmacophore [24], and binding affinity analyses [25] would likely enhance the applicability of the drugome approach. Once refined, this approach could be applied to non-MTB bacteria and other computational methods of drug repositioning, given that there is sufficient protein structural information available for the organism of interest. Another potential extension of the TB drugome approach would be to include the metabolic derivatives of FDA-approved drugs. Our results with 4-OHT suggest that this extension may be helpful. Still, structural information regarding the binding of such derivatives with proteins will be required. Since this information remains scarce, searching drug derivatives for potential drug repositioning using the TB drugome approach may yield a fairly low hit rate.

## Materials and Methods

### Construction of the updated TB drugome

The TB drugome used in this study was constructed as described by Kinnings *et al*. [5], using updated protein structural information. Briefly, the 274 FDA-approved human drugs tested in Kinnings *et al.* [5] and 43 additional drugs were retrieved from the DrugBank [26]. Six drugs with no chemical component identifier (CID) in the RCSB PDB (www.rcsb.org) were discarded. Thus, we obtained 311 drugs (Table S7), which corresponded to 321 CIDs in PDB (10 drugs had two CIDs). Next, 1,729 entries of protein-chemical interactions involving these CIDs were extracted from the PDB at ligand-expo.rcsb.org. In addition, 1,215 MTB structural entries were retrieved from the PDB (as of January 2013) and cross-referenced to 405 UniProt (http://www.uniprot.org/) MTB proteins.

Structural similarities between the drug target and MTB proteins were evaluated using the SMAP program with the customized parameters suggested by the developer [27]. The rationale was that proteins with similar ligand-binding sites could potentially be targeted by the same drugs. Based on the SMAP analysis, we identified MTB proteins that were potentially targeted by the human drugs included in the drugome screen and ranked the drugs according to the number of MTB proteins that were potentially targeted. We selected 23 drugs (in addition to RIF) for experimental validation (Table 1). Fifteen of these drugs were the top 15 drugs from Kinning et al.'s results [5]. One (tamoxifen) was an FDA-approved drug, of which a derivative (4-OHT) was a top-ranked chemical in the TB drugome but not an approved drug. Other drugs were randomly selected from the 311 drugs.

### Anti-mycobacterial activity of individual drugs

The H37Rv (ATCC 27294) and H37Ra (ATCC 25177) strains of MTB were used to evaluate drug efficacy. These strains were cultured at 37°C in Middlebrook 7H9 medium, supplemented with 10% (vol/vol) OADC enrichment (0.85% NaCl, 5% bovine serum albumin [BSA] fraction V, 2% D-dextrose, 0.004% catalase, and 0.05% oleic acid), 0.2% (vol/vol) glycerol, and 0.05% (vol/vol) Tween 80, to an optical density at 600 nm (OD_600_) of approximately 0.6–0.8. The cultures were subsequently diluted with supplemented Middlebrook 7H9 medium to 1×10^6^ cells/mL for use in the assay. The 7H9 broth with Tween 80 was used as the subculture medium for *Mycobacterium* species and in the preparation of inocula for drug susceptibility testing. All drugs and chemicals used in this study were purchased from Sigma-Aldrich.

All of the tested drugs were dissolved in DMSO. Bacteria were treated with four concentrations (2.5, 5, 10, and 20 mg/L) of drugs and incubated at 37°C for 7 days (H37Ra) or 14 days (H37Rv). We established an upper limit of 20 mg/L because most of the first- and second-line anti-TB drugs have an MIC lower than this concentration [28]. RIF was used as a positive control. Inhibitory effects of the drugs were evaluated with reference to the OD_600_ of the MTB culture. The OD_600_ value that indicated <X% (e.g., 2%) growth compared to the no-drug control was confirmed by counting the diluted cell culture. Absorbance values were normalized to the drug-free control samples. Three replicates were conducted for each drug treatment to derive standard deviations.

### Anti-mycobacterial activity of drug combinations

Three concentrations below the MIC (∼20 mg/L) of 4-OHT and tamoxifen (5, 10, and 20 mg/L) were tested separately in combination with the first-line TB drugs, RIF, INH, and EMB. H37Ra cultures were treated with various drug combinations and incubated at 37°C for 7 days. Efficacy values of the combination drug treatments were evaluated by the OD_600_ of the MTB culture. The absorbance values were normalized and standard deviations were derived as described above.

## Supporting Information

Figure S1 **Distribution of the number of potential MTB protein targets for 217 FDA-approved drugs.**
(TIF)

Figure S2 **Growth of** ***M. tuberculosis*** **H37Ra at different concentrations of RIF.** Arrows indicate the concentrations tested in this study.
(TIF)

Figure S3 **Assessing the congruence between the OD_600_ method and ELISA-based measurements of MTB growth.** (A) MTB growth curves based on OD_600_ (left column) and ELISA measurements (right column) at different concentrations of INH (*upper half*) and EMB (*lower half);* (B) Linear regression results comparing the OD_600_ and ELISA measurements for INH (left) and EMB (right) experiments.
(TIF)

Table S1 Inferred MIC_50_ and MIC_90_ values of the tested drugs for *M. tuberculosis* H37Ra.
(DOCX)

Table S2 Inferred MIC_50_ and MIC_90_ values of the tested drugs for *M. tuberculosis* H37Rv.
(DOCX)

Table S3 Inhibitory effects of 4-OHT in combination with RIF, INH, or EMB on *M. tuberculosis* H37Ra.
(DOCX)

Table S4 Inhibitory effects of tamoxifen in combination with RIF, INH, or EMB on *M. tuberculosis* H37Ra.
(DOCX)

Table S5 Predicted molecular targets of 4-OHT.
(XLSX)

Table S6 Predicted molecular targets of rifampin.
(XLSX)

Table S7 List of approved human drugs included in the drugome screen approach.
(XLS)
