# Supplementary figures and images for: Pros and Cons of the Tuberculosis Drugome Approach – An Empirical Analysis
Source: PLoS One. 2014 Jun 27;9(6):e100829. doi: 10.1371/journal.pone.0100829 (PMC4074101; doi:10.1371/journal.pone.0100829)

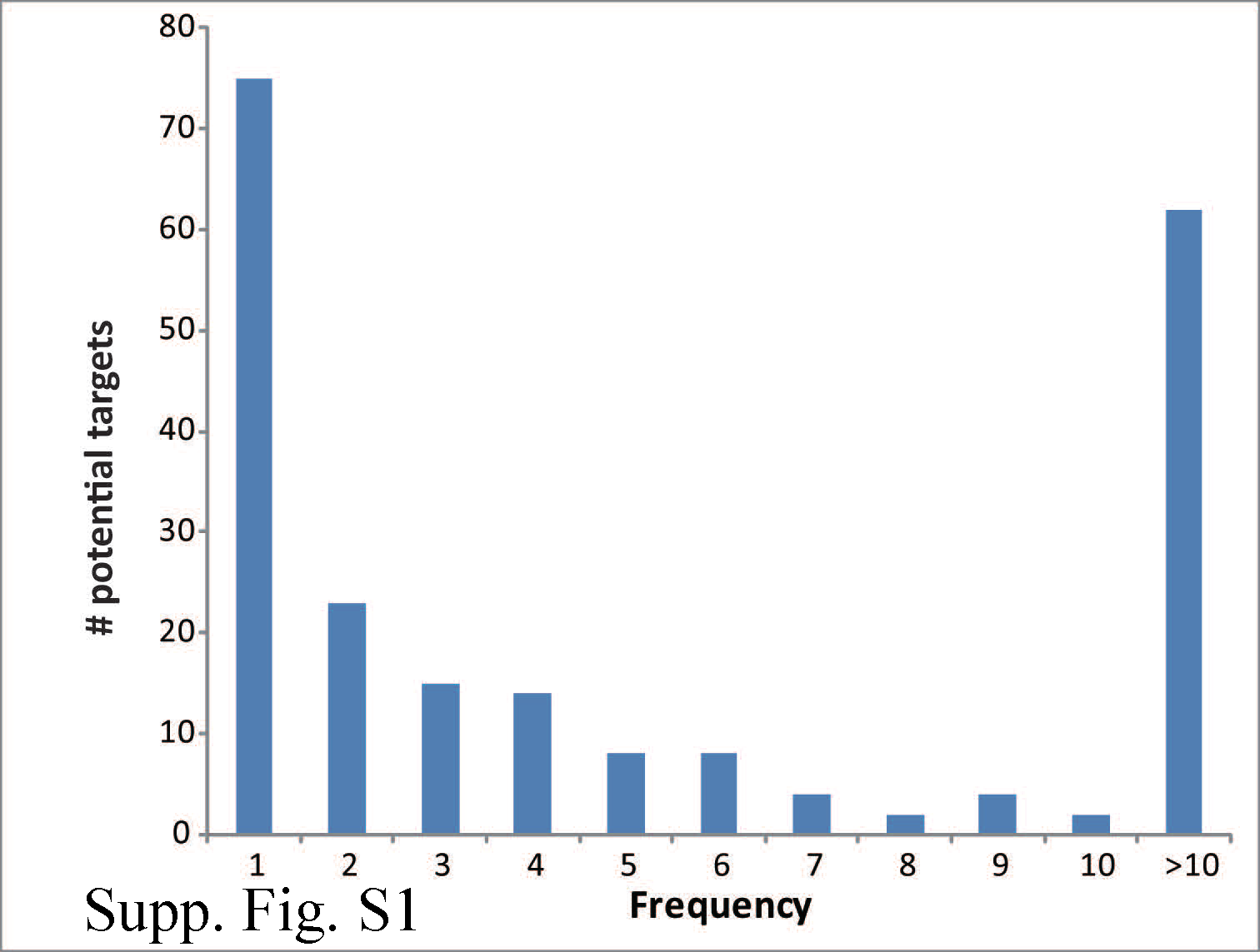

Supplement: Figure S1 — Distribution of the number of potential MTB protein targets for 217 FDA-approved drugs. (TIF) [file pone.0100829.s001.tif]

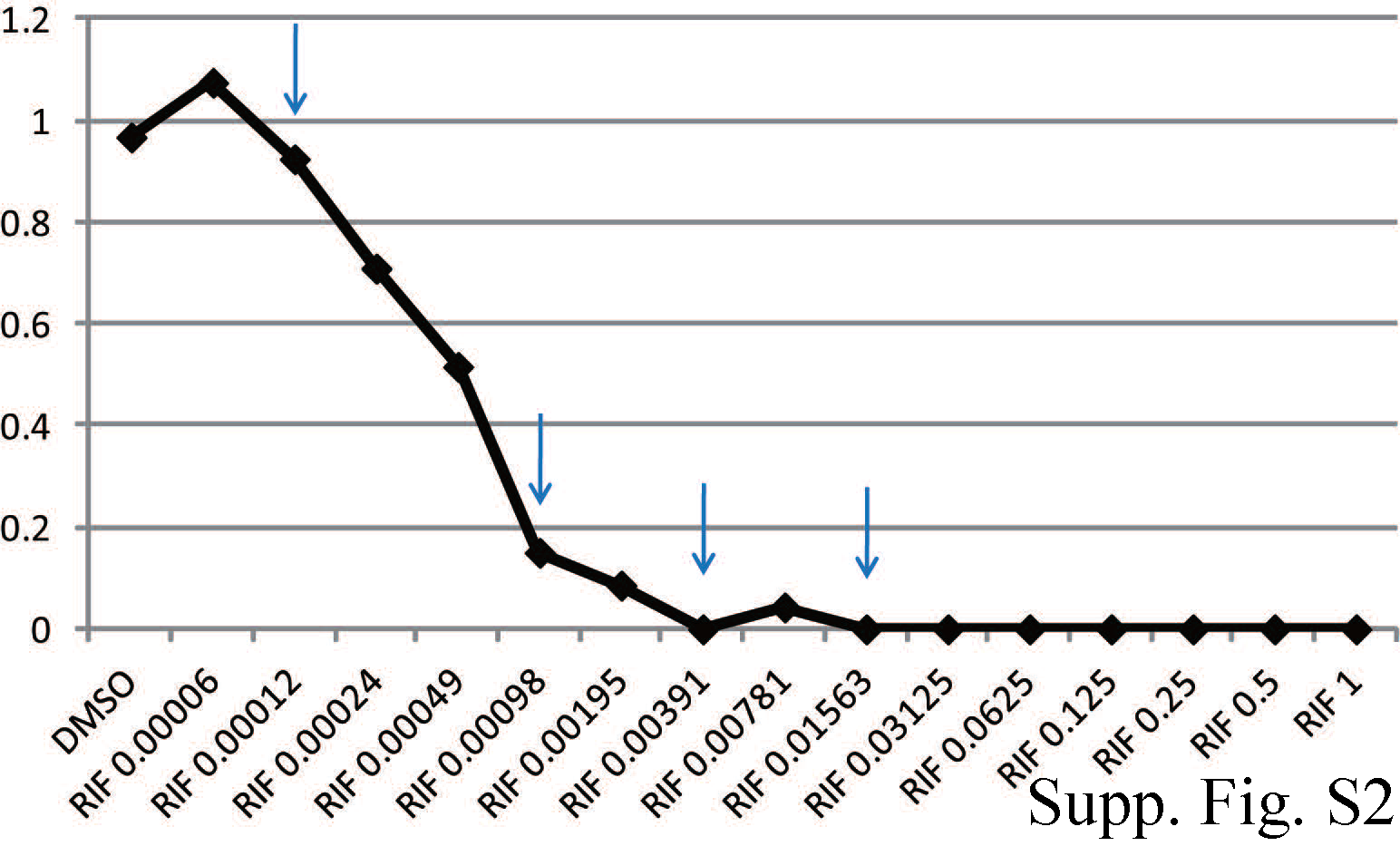

Supplement: Figure S2 — Growth of M. tuberculosis H37Ra at different concentrations of RIF. Arrows indicate the concentrations tested in this study. (TIF) [file pone.0100829.s002.tif]

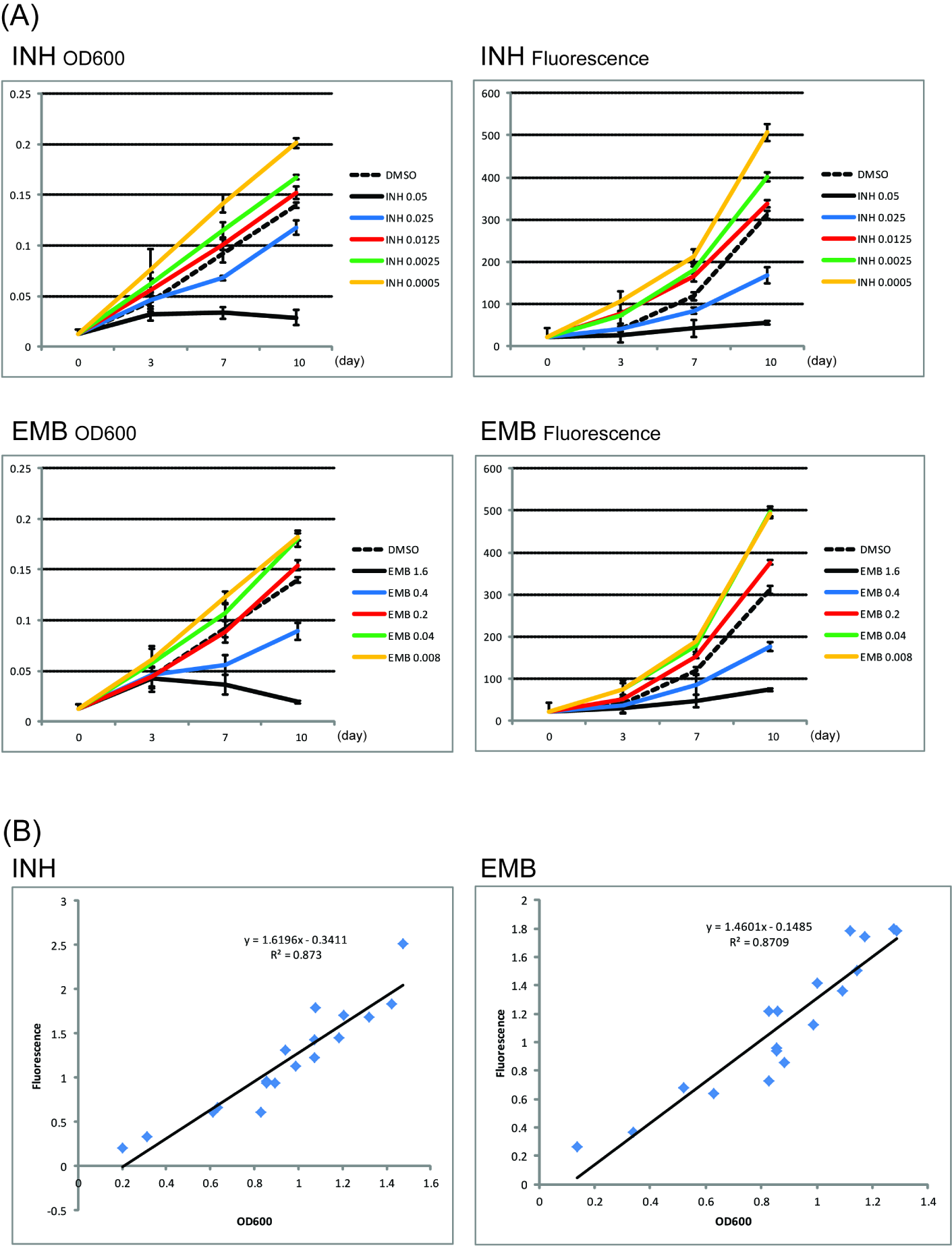

Supplement: Figure S3 — Assessing the congruence between the OD600 method and ELISA-based measurements of MTB growth. (A) MTB growth curves based on OD600 (left column) and ELISA measurements (right column) at different concentrations of INH (upper half) and EMB (lower half); (B) Linear regression results comparing the OD600 and ELISA measurements for INH (left) and EMB (right) experiments. (TIF) [file pone.0100829.s003.tif]
