# Supplementary material for: Pros and Cons of the Tuberculosis Drugome Approach – An Empirical Analysis
Source: PLoS One. 2014 Jun 27;9(6):e100829. doi: 10.1371/journal.pone.0100829 (PMC4074101; doi:10.1371/journal.pone.0100829)
Supplement: Table S1 — Inferred MIC50 and MIC90 values of the tested drugs for M. tuberculosis H37Ra. (DOCX) [file pone.0100829.s004.docx]

| Supplementary Table S1. Absorbance and Bacteriostasis activity for *M. tuberculosis* H37Ra | | | | | | | | | | | | | | | | | |
| --- | --- | --- | --- | --- | --- | --- | --- | --- | --- | --- | --- | --- | --- | --- | --- | --- | --- |
|  | Absorbance normalized to  the untreated control , mean (SD) | | | | | | | |  | Bacteriostasis activity ( ﹪) | | | |  | Minimum Inhibitory Concentration | |  |
|  |  |  |  |  |  |  |  |  |  |  |  |  |  |  |  |  |  |
| Compound **\** mg/L | 20 |  | 10 |  | 5 |  | 2.5 |  |  | 20 | 10 | 5 | 2.5 |  | MIC_90_ | MIC_50_ |  |
| 01. Alitretinoin | 0.723 | (0.141) | 0.890 | (0.096) | 0.999 | (0.043) | 1.007 | (0.035) |  | 27.7 | 11.0 | 0.1 | -0.7 |  | ND^1^ | ND |  |
| 02. Levothyroxine | 0.753 | (0.035) | 0.837 | (0.072) | 0.948 | (0.019) | 0.976 | (0.018) |  | 24.7 | 16.3 | 5.2 | 2.4 |  | ND | ND |  |
| 03. Methotrexate | 0.763 | (0.006) | 0.898 | (0.086) | 0.921 | (0.102) | 0.936 | (0.034) |  | 23.7 | 10.2 | 7.9 | 6.4 |  | ND | ND |  |
| 04. Estradiol | 0.665 | (0.047) | 0.723 | (0.054) | 0.864 | (0.084) | 0.974 | (0.036) |  | 33.5 | 27.7 | 13.6 | 2.6 |  | ND | ND |  |
| 05. Tamoxifen | 0.134 | (0.058) | 0.422 | (0.083) | 0.613 | (0.073) | 0.765 | (0.057) |  | 86.6 | 57.8 | 38.7 | 23.5 |  | ND | 5~10 |  |
| 06. 4-hydroxytamoxifen | 0.064 | (0.007) | 0.218 | (0.008) | 0.483 | (0.114) | 0.688 | (0.046) |  | 93.6 | 78.2 | 51.7 | 31.2 |  | 10~20 | 2.5~5 |  |
| 07. Amantadine | 0.693 | (0.097) | 0.776 | (0.054) | 0.955 | (0.069) | 1.025 | (0.015) |  | 30.7 | 22.4 | 4.5 | -2.5 |  | ND | ND |  |
| 08. Raloxifene | 0.409 | (0.022) | 0.557 | (0.018) | 0.683 | (0.101) | 0.822 | (0.043) |  | 59.1 | 44.3 | 31.7 | 17.8 |  | ND | 10~20 |  |
| 09. Propofol | 0.793 | (0.134) | 0.864 | (0.119) | 0.915 | (0.038) | 0.980 | (0.009) |  | 20.7 | 13.6 | 8.5 | 2.0 |  | ND | ND |  |
| 10. Indinavir | 0.839 | (0.031) | 0.894 | (0.035) | 0.899 | (0.098) | 0.924 | (0.072) |  | 16.1 | 10.6 | 10.1 | 7.6 |  | ND | ND |  |
| 11. Ritonavir | 0.359 | (0.130) | 0.519 | (0.150) | 0.743 | (0.056) | 0.902 | (0.046) |  | 64.1 | 48.1 | 25.7 | 9.8 |  | ND | 10~20 |  |
| 12. Darunavir | 0.829 | (0.079) | 0.837 | (0.110) | 0.859 | (0.135) | 0.923 | (0.091) |  | 17.1 | 16.3 | 14.1 | 7.7 |  | ND | ND |  |
| 13. Lopinavir | 0.166 | (0.107) | 0.335 | (0.127) | 0.513 | (0.144) | 0.749 | (0.040) |  | 83.4 | 66.5 | 48.7 | 25.1 |  | ND | 5~10 |  |
| 14. Penicillamine | 0.951 | (0.048) | 0.960 | (0.054) | 0.947 | (0.063) | 0.934 | (0.068) |  | 4.9 | 4.0 | 5.3 | 6.6 |  | ND | ND |  |
| 15. Nelfinavir | 0.371 | (0.142) | 0.450 | (0.127) | 0.660 | (0.063) | 0.842 | (0.071) |  | 62.9 | 55.0 | 34.0 | 15.8 |  | ND | 5~10 |  |
| 16. Dexamethasone | 0.912 | (0.097) | 0.998 | (0.036) | 0.941 | (0.091) | 0.954 | (0.049) |  | 8.8 | 0.2 | 5.9 | 4.6 |  | ND | ND |  |
| 17. Fluconazole | 0.607 | (0.100) | 0.746 | (0.033) | 0.872 | (0.038) | 0.928 | (0.089) |  | 39.3 | 25.4 | 12.8 | 7.2 |  | ND | ND |  |
| 18. Trimethoprim | 0.923 | (0.038) | 0.959 | (0.069) | 0.951 | (0.049) | 0.965 | (0.052) |  | 7.7 | 4.1 | 4.9 | 3.5 |  | ND | ND |  |
| 19. Cytarabine | 0.585 | (0.057) | 0.771 | (0.019) | 0.836 | (0.036) | 0.976 | (0.052) |  | 41.5 | 22.9 | 16.4 | 2.4 |  | ND | ND |  |
| 20. Spironolactone | 0.832 | (0.069) | 0.906 | (0.052) | 0.879 | (0.069) | 0.917 | (0.023) |  | 16.8 | 9.4 | 12.1 | 8.3 |  | ND | ND |  |
| 21. Indomethacin | 0.625 | (0.062) | 0.773 | (0.103) | 0.927 | (0.118) | 0.958 | (0.023) |  | 37.5 | 22.7 | 7.3 | 4.2 |  | ND | ND |  |
| 22. Liothyronine | 0.745 | (0.075) | 0.834 | (0.102) | 0.904 | (0.130) | 0.955 | (0.139) |  | 25.5 | 16.6 | 9.6 | 4.5 |  | ND | ND |  |
| 23. Progesterone | 0.305 | (0.053) | 0.566 | (0.014) | 0.791 | (0.060) | 0.843 | (0.077) |  | 69.5 | 43.4 | 20.9 | 15.7 |  | ND | 10~20 |  |

^1^ND, not determined because the MIC is beyond the maximal tested concentration (20mg/L).
