# Supplementary material for: Pros and Cons of the Tuberculosis Drugome Approach – An Empirical Analysis
Source: PLoS One. 2014 Jun 27;9(6):e100829. doi: 10.1371/journal.pone.0100829 (PMC4074101; doi:10.1371/journal.pone.0100829)
Supplement: Table S2 — Inferred MIC50 and MIC90 values of the tested drugs for M. tuberculosis H37Rv. (DOCX) [file pone.0100829.s005.docx]

| Supplementary Table S2. Absorbance and Bacteriostasis activity for *M. tuberculosis* H37Rv | | | | | | | | | | | | | | | | | |
| --- | --- | --- | --- | --- | --- | --- | --- | --- | --- | --- | --- | --- | --- | --- | --- | --- | --- |
|  | Absorbance normalized to  the untreated control , mean (SD) | | | | | | | |  | Bacteriostasis activity ( ﹪) | | | |  | Minimum Inhibitory Concentration | |  |
|  |  |  |  |  |  |  |  |  |  |  |  |  |  |  |  |  |  |
| Compound **\** mg/L | 20 |  | 10 |  | 5 |  | 2.5 |  |  | 20 | 10 | 5 | 2.5 |  | MIC_90_ | MIC_50_ |  |
| 01. Alitretinoin | 0.857 | (0.063) | 0.993 | (0.216) | 1.010 | (0.123) | 1.030 | (0.151) |  | 14.3 | 0.7 | -1.0 | -3.0 |  | ND^1^ | ND |  |
| 02. Levothyroxine | 1.047 | (0.270) | 1.075 | (0.116) | 1.149 | (0.222) | 1.115 | (0.115) |  | -4.7 | -7.5 | -14.9 | -11.5 |  | ND | ND |  |
| 03. Methotrexate | 0.513 | (0.142) | 0.761 | (0.062) | 0.842 | (0.020) | 0.980 | (0.041) |  | 48.7 | 23.9 | 15.8 | 2.0 |  | ND | ND |  |
| 04. Estradiol | 0.989 | (0.364) | 1.021 | (0.241) | 1.101 | (0.171) | 1.024 | (0.198) |  | 1.1 | -2.1 | -10.1 | -2.4 |  | ND | ND |  |
| 05. Tamoxifen | 0.354 | (0.137) | 0.705 | (0.218) | 0.724 | (0.090) | 0.854 | (0.058) |  | 64.6 | 29.5 | 27.6 | 14.6 |  | ND | 10~20 |  |
| 06. 4-hydroxytamoxifen | 0.049 | (0.030) | 0.282 | (0.062) | 0.680 | (0.146) | 0.967 | (0.142) |  | 95.1 | 71.8 | 32.0 | 3.3 |  | 10~20 | 5~10 |  |
| 07. Amantadine | 0.961 | (0.113) | 0.942 | (0.129) | 0.911 | (0.077) | 0.956 | (0.147) |  | 3.9 | 5.8 | 8.9 | 4.4 |  | ND | ND |  |
| 08. Raloxifene | 0.957 | (0.216) | 1.058 | (0.254) | 1.152 | (0.231) | 1.131 | (0.144) |  | 4.3 | -5.8 | -15.2 | -13.1 |  | ND | ND |  |
| 09. Propofol | 1.027 | (0.143) | 1.019 | (0.047) | 1.065 | (0.017) | 1.078 | (0.093) |  | -2.7 | -1.9 | -6.5 | -7.8 |  | ND | ND |  |
| 11. Ritonavir | 0.687 | (0.154) | 0.803 | (0.241) | 0.851 | (0.153) | 0.912 | (0.117) |  | 31.3 | 19.7 | 14.9 | 8.8 |  | ND | ND |  |
| 12. Darunavir | 0.826 | (0.046) | 0.874 | (0.038) | 0.980 | (0.101) | 0.934 | (0.047) |  | 17.4 | 12.6 | 2.0 | 6.6 |  | ND | ND |  |
| 13. Lopinavir | 0.342 | (0.148) | 0.489 | (0.049) | 0.679 | (0.050) | 0.859 | (0.036) |  | 65.8 | 51.1 | 32.1 | 14.1 |  | ND | 5~10 |  |
| 15. Nelfinavir | 0.764 | (0.210) | 0.879 | (0.117) | 0.933 | (0.124) | 0.886 | (0.041) |  | 23.6 | 12.1 | 6.7 | 11.4 |  | ND | ND |  |
| 17. Fluconazole | 0.897 | (0.060) | 0.955 | (0.118) | 0.856 | (0.051) | 0.891 | (0.076) |  | 10.3 | 4.5 | 14.4 | 10.9 |  | ND | ND |  |
| 19. Cytarabine | 0.769 | (0.049) | 0.819 | (0.044) | 0.858 | (0.099) | 0.896 | (0.049) |  | 23.1 | 18.1 | 14.2 | 10.4 |  | ND | ND |  |
| 21. Indomethacin | 0.738 | (0.223) | 0.781 | (0.221) | 0.928 | (0.170) | 0.920 | (0.100) |  | 26.2 | 21.9 | 7.2 | 8.0 |  | ND | ND |  |
| 22. Liothyronine | 0.848 | (0.233) | 0.918 | (0.205) | 0.948 | (0.173) | 0.972 | (0.180) |  | 15.2 | 8.2 | 5.2 | 2.8 |  | ND | ND |  |
| 23. Progesterone | 0.689 | (0.119) | 0.743 | (0.204) | 0.889 | (0.107) | 0.917 | (0.044) |  | 31.1 | 25.7 | 11.1 | 8.3 |  | ND | ND |  |

^1^ND, not determined because the MIC is beyond the maximal tested concentration (20mg/L).
