# Supplementary material for: Pros and Cons of the Tuberculosis Drugome Approach – An Empirical Analysis
Source: PLoS One. 2014 Jun 27;9(6):e100829. doi: 10.1371/journal.pone.0100829 (PMC4074101; doi:10.1371/journal.pone.0100829)
Supplement: Table S3 — Inhibitory effects of 4-OHT in combination with RIF, INH, or EMB on M. tuberculosis H37Ra. (DOCX) [file pone.0100829.s006.docx]

| Supplementary Table S3. Inhibitory effects of 4-OHT in combination with RIF, INH, or EMB on *M.* *tuberculosis* H37Ra. | | | | | | | | | | | | | | |
| --- | --- | --- | --- | --- | --- | --- | --- | --- | --- | --- | --- | --- | --- | --- |
|  | Absorbance normalized to  the untreated control , mean(SD) | | | | | | | |  | Bacteriostasis activity ( ﹪) | | | |  |
|  |  |  |  |  |  |  |  |  |  |  |  |  |  |  |
| First-line drugs **\** 4-OHT | 0 |  | 5 |  | 10 |  | 20 |  |  | 0 | 5 | 10 | 20 |  |
| DMSO | 0.970 | (0.018) | 0.768 | (0.031) | 0.200 | (0.010) | 0.038 | (0.027) |  | 3.0 | 23.2 | 80.0 | 96.2 |  |
| RIF concentration (mg/L) |  |  |  |  |  |  |  |  |  |  |  |  |  |  |
| 0.01562 | 0.023 | (0.040) | 0.008 | (0.008) | 0.000 | (0.000) | 0.000 | (0.000) |  | 97.7 | 99.2 | 100.0 | 100.0 |  |
| 0.00390 | 0.037 | (0.046) | 0.054 | (0.053) | 0.000 | (0.000) | 0.000 | (0.000) |  | 96.3 | 94.6 | 100.0 | 100.0 |  |
| 0.00097 | 0.139 | (0.048) | 0.028 | (0.007) | 0.004 | (0.007) | 0.004 | (0.007) |  | 86.1 | 97.2 | 99.6 | 99.6 |  |
| 0.00012 | 0.900 | (0.035) | 0.661 | (0.094) | 0.096 | (0.105) | 0.009 | (0.008) |  | 10.0 | 33.9 | 90.4 | 99.1 |  |
| INH concentration (mg/L) |  |  |  |  |  |  |  |  |  |  |  |  |  |  |
| 0.05 | 0.009 | (0.008) | 0.016 | (0.005) | 0.004 | (0.007) | 0.000 | (0.000) |  | 99.1 | 98.4 | 99.6 | 100.0 |  |
| 0.025 | 0.242 | (0.132) | 0.025 | (0.005) | 0.000 | (0.000) | 0.000 | (0.000) |  | 75.8 | 97.5 | 100.0 | 100.0 |  |
| 0.0125 | 0.851 | (0.015) | 0.501 | (0.105) | 0.045 | (0.024) | 0.000 | (0.000) |  | 14.9 | 49.9 | 95.5 | 100.0 |  |
| EMB concentration (mg/L) |  |  |  |  |  |  |  |  |  |  |  |  |  |  |
| 1.6 | 0.025 | (0.013) | 0.011 | (0.010) | 0.007 | (0.006) | 0.000 | (0.000) |  | 97.5 | 98.9 | 99.3 | 100.0 |  |
| 0.8 | 0.051 | (0.006) | 0.016 | (0.006) | 0.011 | (0.011) | 0.004 | (0.007) |  | 94.9 | 98.4 | 98.9 | 99.6 |  |
| 0.4 | 0.104 | (0.025) | 0.060 | (0.027) | 0.033 | (0.034) | 0.011 | (0.020) |  | 89.6 | 94.0 | 96.7 | 98.9 |  |
| 0.2 | 0.434 | (0.106) | 0.304 | (0.040) | 0.145 | (0.052) | 0.021 | (0.018) |  | 56.6 | 69.6 | 85.5 | 97.9 |  |
| 0.1 | 0.904 | (0.086) | 0.765 | (0.267) | 0.234 | (0.099) | 0.039 | (0.042) |  | 9.6 | 23.5 | 76.6 | 96.1 |  |
