# Supplementary material for: Pros and Cons of the Tuberculosis Drugome Approach – An Empirical Analysis
Source: PLoS One. 2014 Jun 27;9(6):e100829. doi: 10.1371/journal.pone.0100829 (PMC4074101; doi:10.1371/journal.pone.0100829)
Supplement: Table S4 — Inhibitory effects of tamoxifen in combination with RIF, INH, or EMB on M. tuberculosis H37Ra. (DOCX) [file pone.0100829.s007.docx]

| Supplementary Table S4. Inhibitory effects of tamoxifen in combination with RIF, INH, or EMB on *M.* *tuberculosis* H37Ra.. | | | | | | | | | | | | | | |
| --- | --- | --- | --- | --- | --- | --- | --- | --- | --- | --- | --- | --- | --- | --- |
|  | Absorbance normalized to  the untreated control , mean(SD) | | | | | | | |  | Bacteriostasis activity ( ﹪) | | | |  |
|  |  |  |  |  |  |  |  |  |  |  |  |  |  |  |
| First-line drugs **\** tamoxifen | 0 |  | 5 |  | 10 |  | 20 |  |  | 0 | 5 | 10 | 20 |  |
| DMSO | 0.970 | (0.018) | 0.840 | (0.101) | 0.466 | (0.063) | 0.102 | (0.018) |  | 3.0 | 16.0 | 53.4 | 89.8 |  |
| RIF concentration (mg/L) |  |  |  |  |  |  |  |  |  |  |  |  |  |  |
| 0.01562 | 0.023 | (0.040) | 0.000 | (0.000) | 0.004 | (0.007) | 0.000 | (0.000) |  | 97.7 | 100.0 | 99.6 | 100.0 |  |
| 0.00390 | 0.037 | (0.046) | 0.012 | (0.020) | 0.004 | (0.007) | 0.000 | (0.000) |  | 96.3 | 98.8 | 99.6 | 100.0 |  |
| 0.00097 | 0.139 | (0.048) | 0.076 | (0.068) | 0.009 | (0.008) | 0.000 | (0.000) |  | 86.1 | 92.4 | 99.1 | 100.0 |  |
| 0.00012 | 0.900 | (0.035) | 0.790 | (0.061) | 0.394 | (0.155) | 0.038 | (0.027) |  | 10.0 | 21.0 | 60.6 | 96.2 |  |
| INH concentration (mg/L) |  |  |  |  |  |  |  |  |  |  |  |  |  |  |
| 0.05 | 0.009 | (0.008) | 0.005 | (0.009) | 0.014 | (0.015) | 0.000 | (0.000) |  | 99.1 | 99.5 | 98.6 | 100.0 |  |
| 0.025 | 0.242 | (0.132) | 0.026 | (0.018) | 0.009 | (0.008) | 0.004 | (0.007) |  | 75.8 | 97.4 | 99.1 | 99.6 |  |
| 0.0125 | 0.851 | (0.015) | 0.596 | (0.141) | 0.226 | (0.154) | 0.004 | (0.007) |  | 14.9 | 40.4 | 77.4 | 99.6 |  |
| EMB concentration (mg/L) |  |  |  |  |  |  |  |  |  |  |  |  |  |  |
| 1.6 | 0.025 | (0.013) | 0.030 | (0.014) | 0.023 | (0.008) | 0.019 | (0.024) |  | 97.5 | 97.0 | 97.7 | 98.1 |  |
| 0.8 | 0.051 | (0.006) | 0.033 | (0.018) | 0.033 | (0.011) | 0.028 | (0.026) |  | 94.9 | 96.7 | 96.7 | 97.2 |  |
| 0.4 | 0.104 | (0.025) | 0.134 | (0.077) | 0.111 | (0.039) | 0.041 | (0.019) |  | 89.6 | 86.6 | 88.9 | 95.9 |  |
| 0.2 | 0.434 | (0.106) | 0.404 | (0.061) | 0.262 | (0.085) | 0.070 | (0.021) |  | 56.6 | 59.6 | 73.8 | 93.0 |  |
| 0.1 | 0.904 | (0.086) | 0.896 | (0.192) | 0.518 | (0.184) | 0.166 | (0.121) |  | 9.6 | 10.4 | 48.2 | 83.4 |  |
